# Effect of Cytochrome b5 Content on the Activity of Polymorphic CYP1A2, 2B6, and 2E1 in Human Liver Microsomes

**DOI:** 10.1371/journal.pone.0128547

**Published:** 2015-06-05

**Authors:** Haifeng Zhang, Na Gao, Tingting Liu, Yan Fang, Bing Qi, Qiang Wen, Jun Zhou, Linjing Jia, Hailing Qiao

**Affiliations:** Institute of Clinical Pharmacology, Zhengzhou University, Zhengzhou, China; Indian Institute of Toxicology Reserach, INDIA

## Abstract

Human cytochrome b5 (Cyt b5) plays important roles in cytochrome P450 (CYP)-mediated drug metabolism. However, the expression level of Cyt b5 in normal human liver remains largely unknown. The effect of Cyt b5 on overall CYP activity in human liver microsomes (HLM) has rarely been reported and the relationship between Cyt b5 and the activity of polymorphic CYP has not been systematically investigated. In this study, we found that the median value of Cyt b5 protein was 270.01 pmol/mg from 123 HLM samples, and 12- and 19-fold individual variation was observed in Cyt b5 mRNA and protein levels, respectively. Gender and smoking clearly influenced Cyt b5 content. In addition, we found that Cyt b5 protein levels significantly correlated with the overall activity of CYP1A2, 2B6, and 2E1 in HLM. However, when the CYP activities were sorted by single nucleotide polymorphisms (SNP), the effect of Cyt b5 protein on the kinetic parameters varied greatly. There were significant correlations between Cyt b5 content and V_max_ and CL_int_ of CYP1A2 wild-types (3860GG, 2159GG, and 5347CC) as well as homozygous mutants (163AA and 3113GG). In contrast to V_max_ and CL_int_, the K_m_ of CYP2B6 516GG and 785AA genotypes was inversely associated with Cyt b5 content. Correlations between Cyt b5 content and V_max_ and CL_int_ of CYP2E1 -1293GG, -1293GC, 7632TT, 7632TA, -333TT, and -352AA genotypes were also observed. In conclusion, Cyt b5 expression levels varied considerably in the Chinese cohort from this study. Cyt b5 had significant impact on the overall activity of CYP1A2, 2B6, and 2E1 in HLM and the effects of Cyt b5 protein on polymorphic CYP1A2, 2B6, and 2E1 activity were SNP-dependent. These findings suggest that Cyt b5 plays an important role in CYP-mediated activities in HLM and may possibly be a contributing factor for the individual variation observed in CYP enzyme activities.

## Introduction

Human cytochrome b5 (Cyt b5), a small molecular protein containing one heme prothetic group, is encoded by a single gene on chromosome 18q23 that generates two isoforms through alternative splicing [1,2]. Isoform 1 is a soluble cytoplasmic form found in erythrocytes, while isoform 2 is predominantly bound to the cytoplasmic side of the endoplasmic reticulum in many tissues, such as liver [3,4]. Cyt b5 functions as an electron carrier or an allosteric effector and plays important roles in many aspects of human physiology, including metabolism, hematology and endocrinology, and xenobiotic metabolism [5], which involves cytochrome P450 (CYP)-catalyzed drug metabolism, fatty acid desaturation, methemoglobin reduction, and steroidogenes [6–8].

No report to date has characterized the absolute content of Cyt b5 over a large sampling of normal human livers. Two studies reported the absolute content of Cyt b5 using a spectral method, but these studies only assessed a small number of liver samples [9,10]. Sacco *et al*. reported the relative expression level of Cyt b5 in a large number of livers, but the results were not provided quantitatively [3]. A study by Gan *et al*. provided both spectral and western blot analysis of absolute Cyt b5 content, but the mean value and range measured by these two methods were quite different [11]. To the best of our knowledge, only the study by Gan *et al*. compared the effects of age and gender on microsomal Cyt b5 content, but the results were not clear due to the different methods used. Therefore, a well-designed study to evaluate individual variation and influencing factors in the Cyt b5 absolute content in a large group of human livers is still needed.

Cytochrome P450 (CYP) represents a super family of predominantly liver-localized enzymes that plays critical roles in biology and is responsible for the biotransformation of most xenobiotics, including 70–80% of all drugs currently in clinical use [12,13]. Extensive individual variation is observed the in the metabolic activities catalyzed by CYP, which can result in dramatic differences in the way patients respond to the same drugs, including lack of response, overreaction, or toxicity [14]. Human CYP gene polymorphisms have been intensely investigated and polymorphisms have been reported to be essential factors that control individual differences in the metabolism of drugs [15,16]. CYP1A2 metabolizes approximately 9% of the most widely used drugs and is involved in the oxidative transformation of aromatic amines and heterocyclic compounds. Many alleles and haplotype variants have been defined, and some have been associated with altered expression or code for proteins with altered enzyme activity [13]. CYP2B6 also plays an important role in a number of structurally diverse classes of frequently used drugs, such as nicotine, lidocaine, S-mephenytoin, cyclophosphamide, amitriptyline, and diazepam [13]. Large individual variation exists in CYP2B6 activity and common polymorphisms in the CYP2B6 gene have been found to contribute to variation in CYP2B6 activity [17]. Among various human CYPs, CYP2E1 is of particular interest because of its involvement in the metabolic activation of a large number of toxicants and procarcinogens in addition to metabolizing approximately 3% of all clinical drugs [13,18]. These three CYP enzymes are therefore very important in the metabolism of widely used drugs and activation of environmental procarcinogens.

In the early 1970s, Estabrook *et al*. first proved that b5 enhanced the metabolism of several CYP substrates [19]. Since then several studies have concentrated on this area, but the functional role of Cyt b5 in CYP-mediated oxidation is complex. Depending on the CYP isoform, substrate, and experimental condition, Cyt b5 can stimulate, inhibit, or have no effect on CYP-involved reactions [20]. It is well-accepted that Cyt b5 is required for the 17, 20-lyase activity of bifunctional steroidogenic enzyme CYP17A1 via protein-protein interactions [21]. Previous studies in reconstitution systems have demonstrated that Cyt b5 stimulates CYP3A4, 3A5, 2E1, 2C19, 2C9, 2C8, 2B6, and 2A6 activities [22–25]. In addition, Cyt b5 can stimulate some, but not all, CYP3A4-dependent reactions [26]. In contrast, other studies have shown that Cyt b5 has no influence on CYP1A2- and CYP2D6-catalyzed reactions in reconstituted systems [25,27]. However, conditional Cyt b5 knockout mice exhibit a significant reduction in the metabolism of several drugs, including CYP1A2-mediated phenacetin O-demethylation and CYP2D-mediated metoprolol α-hydroxylation, which indicates that the ability of Cyt b5 to stimulate CYP-mediated metabolism is not an artifact of in vitro biochemical assays [5,28].

To date, almost all studies on Cyt b5 have been performed in heterologous reconstitution expression systems [29]. Although studies using recombinant proteins are highly valuable in assessing the principal effects of Cyt b5 on CYP function, the results are often difficult to extrapolate to an in vivo situation. Human liver microsomes (HLM) have advantages in estimating the potential of Cyt b5 as a source of variation for drug-metabolism activity because they include the proteins in their natural environment. Unfortunately, few studies have assessed the effect of Cyt b5 on CYP in HLM. Moreover, the relationships between Cyt b5 and the activities of polymorphic CYP have not been reported. Therefore, in this study we first characterized the expression variation of Cyt b5 in 123 Chinese livers and then analyzed the effects of Cyt b5 content on the metabolic activities of CYP1A2, 2B6, and 2E1. We also systematically evaluated the impact of Cyt b5 on the activities of polymorphic CYP1A2, 2B6, and 2E1 variants in HLM.

## Materials and Methods

### Human liver samples

Human liver samples were obtained from 123 Chinese patients undergoing hepatic surgery during 2012 and 2014 at the first affiliated hospital of Zhengzhou University, the People’s Hospital of Henan Province and the Tumors’ hospital of Henan Province, respectively (Table 1). The project was approved by the ethics committees of Zhengzhou University and written informed consent was obtained from each patient. The mean age (± standard deviation; SD) was 49.3 ± 10.7 (range: 25–69) years for men and 46.9 ± 9.7 (range: 20–75) years for women. Live tissue samples were placed on ice and transferred to our lab within 30 min of the biopsy. The tissue was washed with ice-cold normal saline and minced on ice. All liver samples were stored in liquid nitrogen for further use. Only normal liver tissue was collected, which was confirmed by a liver function test, histopathological analysis, and imaging (ultrasonography or compute tomography).

**Table 1:** Donor characteristics of human liver samples (n = 123).

Variables	Group	Number
Gender	Male	40
	Female	83
Age (years)	20–45	48
	46–60	61
	61–75	14
Smoking	Non-smoking	110
	smoking	13
Drinking	Non-drinking	110
	drinking	13
Medical Diagnosis	cavernous hemangioma of liver	90
	metastatic carcinoma	10
	cholelithiasis	9
	gallbladder cancer	4
	cholangiocellular carcinoma	6
	hepatocellular carcinoma	4
Drug exposure	All patients only used routine anesthetics and had no history of exposure to known CYP-inducing or -inhibiting agents.	

### Preparation of human liver microsomes

Tissue samples were thawed on ice and weighed. The samples were finely homogenized on ice using a glass homogenizer in 0.05 M Tris-HCl (pH 7.0) buffer containing 1.12% KCl (w/v) and 1.12% EDTA (v/v). After mixing, the homogenate was centrifuged at 9,000 x g for 20 min at 4°C. The supernatant was collected and centrifuged at 100,000 x g for 1 h at 4°C with a Beckman Optima L-100K ultracentrifuge. The resulting microsomal pellet was resuspended in 0.15 M Tris-HCl (pH 7.6) buffer and centrifuged for an additional hour at 100,000 x g at 4°C. The final microsome pellet was suspended in 0.25 M sucrose (2 ml per gram of original sample). The microsomal suspension was frozen in liquid nitrogen and stored at -80°C until analysis. Microsomal protein contents were determined according to the Bradford method.

### Quantitation of Cyt b5 protein content

The Cyt b5 content in HLM was determined by calculating the difference in spectra between NADH-reduced and oxidized microsomes in 96-well plates [11]. A total of 200 μl of diluted HLM (1 mg protein/ ml, pH 7.4) were reduced by 5 μl of 10 mM NADH (Solarbio Science and Technology Co. Ltd; China). The differences in spectra from NADH-reduced samples were recorded at a wavelength of 410 to 425 nm using a SpectraMax M2 Multi-detection microplate reader. The determination of Cyt b5 content was based on the absorbance difference between 410 and 425 nm using an extinction coefficient of 185 mM^-1^cm^-1^.

### Measurement of Cyt b5 mRNA levels

Total RNA was extracted from the human liver samples using the Takara RNAiso Plus kit (Takara, Japan). RNA content and its purity were determined using a NanoDrop 2000 (Thermo Fisher Scientific, Waltham, MA). The cDNA for qRT-PCR was synthesized from 1 μg total RNA using the PrimeScript RT reagent kit with gDNA Eraser (Perfect Real Time; Takara, Japan) according to the manufacturer's instructions.

Primers for real-time PCR amplification of the Cyt b5 and Glyceraldehyde-3-phosphate dehydrogenase (GAPDH) were designed by the Takara Company. The forward and reverse primers for Cyt b5 and GADPH were as follows: 5′-CCGTCGCCTTGATGTATCG and 5′- TGGCTTCTTTTCTCCCGTGT; and 5′-AACAGGGTGGTGGACCTCA and 5′-GGAGGGGAGATTCAGTGTGG, respectively. Amplification and fluorescence detection were performed using the ABI 7500 Fast Real-Time PCR system (Applied Biosystems). Real-time PCR amplification was carried out in a mixture (20 μl) containing 2 μl of synthesized cDNA, 10 μl of 2×SYBR Premix Ex Taq II Mix, 0.8 μl (0.4 μM) of each primer, 0.4 μl ROX Reference Dye II, and 6 μl ddH_2_O (SYBR, Premix Ex Taq II, Takara). Cycling conditions consisted of one cycle at 95°C for 30 s followed by 40 cycles at 60°C for 34 s. GAPDH was used a reference gene and the relative expression level of Cyt b5 mRNA was calculated using the 2^-ΔCT^ method (ΔCT equals the difference between Cyt b5 and GADPH).

### Genotypes of CYP1A2, 2B6, and 2E1

Polymorphisms in CYP1A2, 2B6, and 2E1 with frequencies of > 1% in the Chinese population were genotyped in this study sample. Five single nucleotide polymorphisms (SNPs) for CYP1A2 (-3860G>A, -3113G>A, -163C>A, 2159G>A, and 5347C>T), two for CYP2B6 (516 G>T and 785 A>G), and four for CYP2E1 (-1293G>C, -352A>G, -333T>A, and 7632T>A) were detected and the frequencies were 11.4–90.0% (detailed data are publishing elsewhere).

### Determination of CYP1A2, 2B6, and 2E1 activity in HLM

Marker activities selective for CYP1A2, 2B6, and 2E1 were determined in individual assays by incubating 0.3 mg microsomal protein/ml for CYP1A2 and CYP2E1 or 0.5 mg microsomal protein/ml for CYP2B6 with 1 mM NADPH and seven or eight substrate concentrations of substrate (6.25–800 μM for phenacetin, 7.8–500 μM for bupropion, and 7.8–1000 μM for chlorzoxazone). The mixture was pre-incubated for 5 min at 37°C. Optimal incubation times for each substrate were as follows: 30 min for phenacetin O-deethylation and chlorzoxazone 6-hydroxylation and 60 min for bupropion 1-hydroxylation. Incubation conditions ensured linear metabolite formation with respect to reaction time and protein content. Each reaction was terminated after the specified incubation period by adding 20 μl ice-cold acetonitrile or 1 ml ethyl acetate or perchloric acid. Metabolite concentrations were then determined by HPLC-UV or HPLC-FLD. The K_m_ and V_max_ of each HLM were determined by nonlinear regression analysis using GraphPad Prism 5. The CL_int_ was calculated from the ratio of V_max_ to K_m_.

### Correlations between Cyt b5 content and CYP1A2, 2B6, and 2E1 activity

The kinetic analysis for phenacetin O-deethylation, bupropion 1-hydroxylation, and chlorzoxazone 6-hydroxylation in 105 HLM showed that there were 7–38, 12–33, and 21–40-fold variations in K_m_, V_max_, and Cl_int_ of CYP1A2, 2B6, and 2E1, respectively. Only CYP2B6 polymorphisms had significant effects on enzyme activity (detailed data are publishing elsewhere). In this study, we used simple linear regression approaches to evaluate the possible contribution of Cyt b5 levels on the activity of CYP1A2, 2B6, and 2E1. In addition, the effects of Cyt b5 on the activities of polymorphic CYP1A2, 2B6, and 2E1 were also analyzed.

### Statistical Analysis

The normality of the data distribution was checked using Kolmogorov-Smirnov and Shapiro-Wilk methods. Since most data sets were not normally distributed, nonparametric methods were generally used for statistical analyses. The Mann-Whitney U test was used for pairwise comparison and the Kruskal-Wallis H test was used for multiple pairwise comparisons. Non-parametric Spearman rank correlation analysis was performed to calculate the correlation coefficient (r). A P value < 0.05 was considered statistically significant (two-tailed). SPSS statistics 17 software was used for data management and statistical analyses. Graphs were generated using GraphPad Prism software version 5.04.

## Results

### mRNA and protein levels of Cyt b5 in human liver

#### Cyt b5 mRNA

**Fig 1:**
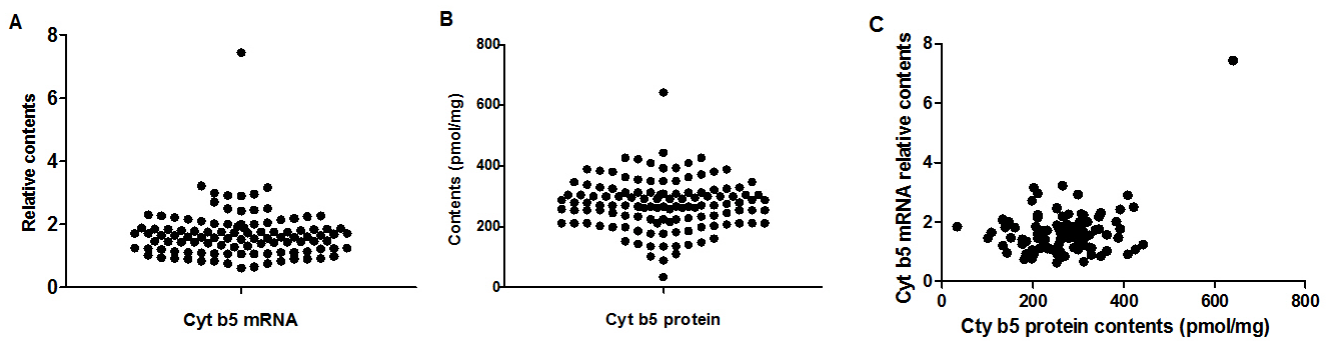
Scatterplot of Cyt b5 mRNA levels (as measured by qPCR, relative to GAPDH, n = 107) (A) and Cyt b5 protein contents (as measured by spectral method, n = 123) (B). Scatterplot of Cyt b5 protein content versus corresponding Cyt b5 mRNA levels (n = 87) (C). The data are presented as the means of three independent experiments.

The Cyt b5 mRNA levels were determined by real-time RT-PCR and normalized to GAPDH mRNA levels (Fig 1A). The relative level of Cyt b5 mRNA in 107 liver tissue samples ranged from 0.61 to 7.44 with a median of 1.59. Among all individuals, Cyt b5 transcript levels were not normally distributed and varied by 12-fold. The variation at the 95% percentage interval (PI) was 4-fold. As shown in Fig 1A, there was one liver sample that had an extremely high Cyt b5 mRNA level (7.44). No unique characteristics were identified for this patient, and therefore it can be inferred that individuals with extreme Cyt b5 mRNA levels exist in the Chinese population.

#### Cyt b5 protein content

Similar to Cyt b5 mRNA, the protein content of Cyt b5 in 123 liver samples was also not normally distributed, with a median of 270.01 pmol/mg. The lowest and highest contents of Cyt b5 protein were 33.75 and 641.27 pmol/mg, respectively, displaying a 19-fold variation (Fig 1B). Consistent with the Cyt b5 mRNA findings, the variation in Cyt b5 protein levels at 95% PI was also 4-fold. Two samples exhibited extreme Cyt b5 protein contents (33.75 and 641.27 pmol/mg, respectively). Interestingly, the same sample with the high Cyt b5 mRNA expression level also exhibited the highest Cyt b5 protein content.

#### The relationship between Cyt b5 mRNA and protein levels

Although the variations in fold change were similar, no correlation was observed between the Cyt b5 mRNA and protein levels (Fig 1C).

#### Effects of demographic factors on Cyt b5 mRNA and protein levels

**Fig 2:**
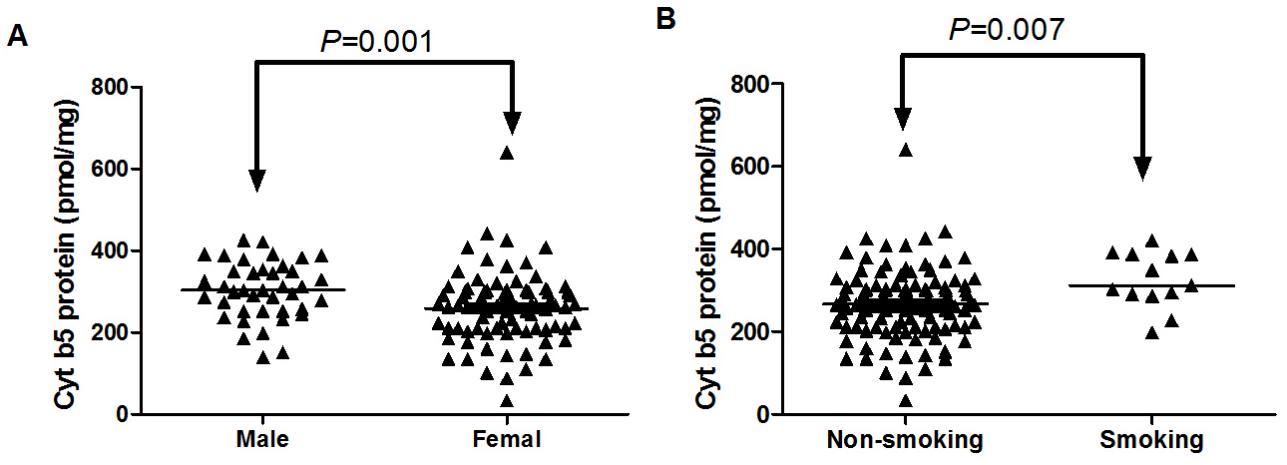
Effects of sex (A) and smoking (B) on the Cyt b5 protein levels in HLM (n = 123). The black horizontal line represents the median value.

To determine whether demographic factors affected the Cyt b5 expression levels in liver samples, Cyt b5 mRNA and protein levels were compared among different demographic parameters (gender, age, smoking status, alcohol use, and disease history). As shown in Fig 2A, Cyt b5 protein contents were significantly higher in males than in females (*P* = 0.001). Statistically significant differences were also observed between smokers and nonsmokers (*P* = 0.007) (Fig 2B). The other demographic factors had no noticeable effect on Cyt b5 protein levels. In addition, no differences in Cyt b5 mRNA levels were detected for the demographic parameters assessed.

### The influence of Cyt b5 on the activity of CYP1A2, 2B6, and 2E1 in HLM

**Fig 3:**
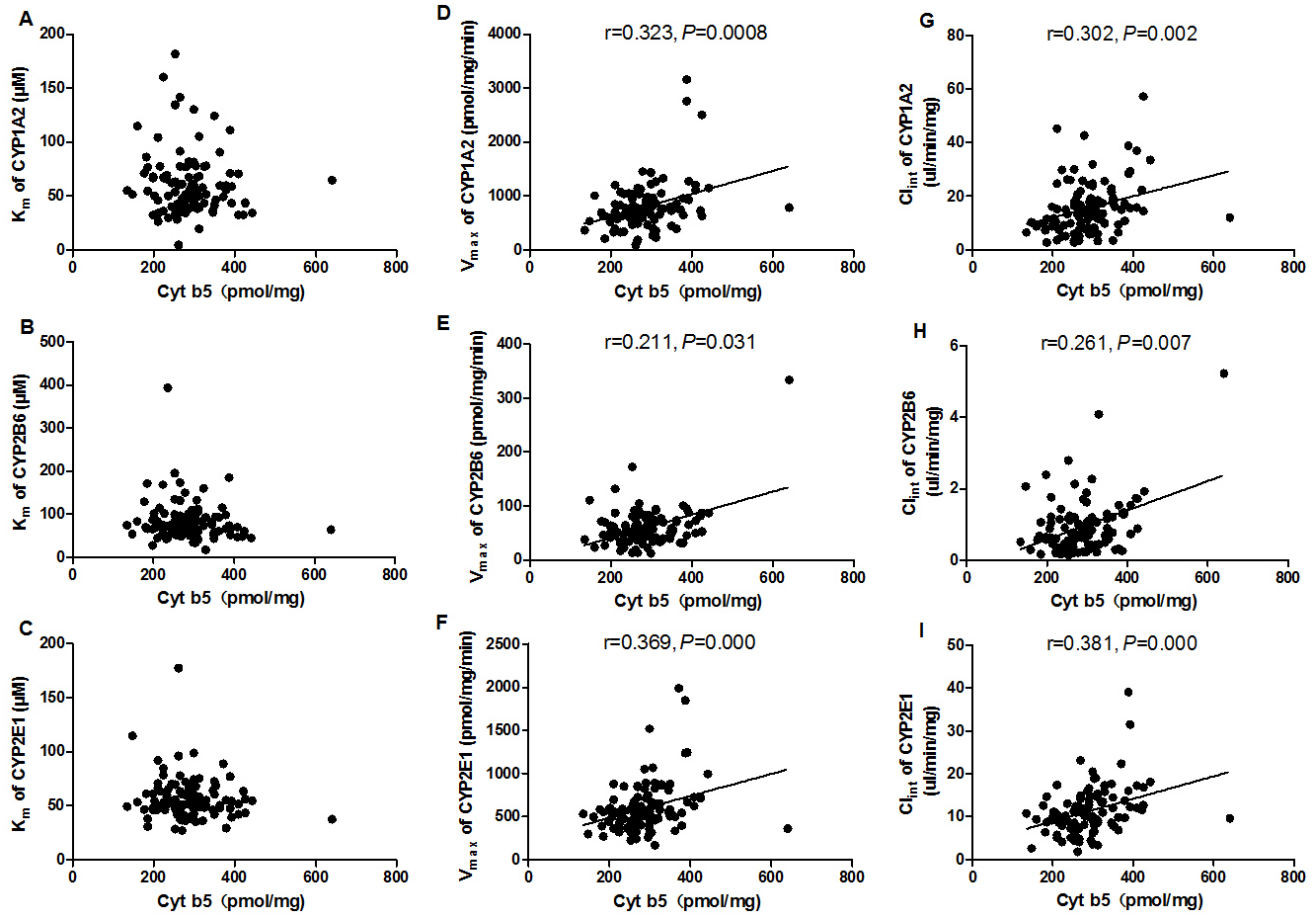
The effects of Cyt b5 content on the overall activity of CYP1A2, 2B6, and 2E1 in a panel of 105 HLM. A, B, C: Correlations between Cyt b5 content and K_m_ of CYP1A2, 2B6, and 2E1. D, E, F: Correlations between Cyt b5 content and V_max_ of CYP1A2, 2B6, and 2E1. G, H, I: Correlations between Cyt b5 content and Cl_int_ of CYP1A2, 2B6, and 2E1.

To assess the potential effect of Cyt b5 on catalytic properties of CYP, the correlation between Cyt b5 content and kinetic parameters for CYP1A2, 2B6, and 2E1 was analyzed. Cyt b5 content was significantly correlated with the V_max_ and CL_int_ of CYP1A2 (phenacetin O-deethylation), 2B6 (bupropion 1-hydroxylation), and 2E1 (chlorzoxazone 6-hydroxylation) activities in 105 individual HLM. However, no significant relationship was observed with the K_m_ of the 3 CYPs (Fig 3).

### The influence of Cyt b5 on the activity of polymorphic CYP1A2, 2B6, and 2E1 in HLM

We next assessed the relationship between Cyt b5 content and the activity of polymorphic CYP1A2, 2B6, and 2E1 (Table 2). Five SNPs in CYP1A2 (-3860G>A, -3113G>A, -163C>A, 2159G>A, and 5347C>T) were used in a correlation analysis. Significant correlations were identified between Cyt b5 content and V_max_ and CL_int_ of CYP1A2 wild-types (-3860GG, 2159GG, and 5347CC). The V_max_ and CL_int_ for homozygous mutant type -163AA and -3113GG also correlated with the Cyt b5 contents. In addition, Cyt b5 contents correlated well with the V_max_ of the -3860G>A mutant heterozygote. However, no correlation was found between the Cyt b5 content and K_m_ of CYP1A2 for all genotypes assessed.

**Table 2:** Associations of Cyt b5 contents with the activity of polymorphic CYP1A2, 2B6, and 2E1.

	Genotype	n	K_m_(r, *P*)	V_max_(r, *P*)	Cl_int_(r, *P*)
CYP1A2		105			
	-3860G>A				
	GG	60	-	0.339(0.008)	0.291(0.024)
	GA	35	-	0.437(0.009)	-
	AA	10	-	-	-
	-163C>A				
	CC	14	-	-	-
	CA	47	-	-	-
	AA	44	-	0.413(0.005)	0.466(0.001)
	5347C>T				
	CC	78	-	0.329(0.003)	0.307(0.006)
	CT	18	-	-	-
	TT	3	-	-	-
	-3113A>G				
	GA	9	-	-	-
	GG	95	-	0.314(0.002)	0.310(0.002)
	2159G >A				
	GG	82	-	0.328(0.003)	0.299(0.006)
	GA	18	-	-	-
	AA	3	-	-	-
CYP2B6					
	516 G>T				
	GG	72	-0.315(0.007)	0.315(0.007)	0.400(0.00)
	GT	32	-	-	-
	TT	1	-	-	-
	785A>G			-	-
	AA	65	-0.327(0.008)	0.343(0.005)	0.430(0.000)
	AG	33	-	-	-
	GG	7	-	-	-
CYP2E1					
	-1293G>C				
	GG	66	-	0.374(0.002)	0.399(0.001)
	GC	31	-	0.408(0.023)	0.376(0.037)
	CC	8	-	-	-
	7632T>A				
	TT	62	-	0.347(0.006)	0.307(0.015)
	TA	32	-	0.350(0.049)	0.429(0.014)
	AA	11	-	-	-
	-333T>A			-	-
	TT	41	-	0.456(0.003)	0.351(0.024)
	TA	44	-	-	-
	AA	20	-0.457(0.043)	0.442(0.051)	0.557(0.011)
	-352A>G				
	AA	71	-	0.377(0.001)	0.292(0.013)
	AG	32	-0.447(0.010)	-	0.482(0.005)
	GG	2	-	-	-

*r*: spearman rank correlation coefficient; ***P*: *P*** value; The black short line represents no correlation.

Similar to CYP1A2, significant correlations were also observed between Cyt b5 contents and V_max_ and CL_int_ of CYP2B6 516GG (516 G>T) and 785AA (785A>G) genotypes. However, the K_m_ values of CYP2B6 516GG and 785AA genotypes were inversely associated with Cyt b5 contents, which was different from that of CYP1A2. For SNPs -1293G>C and 7632T>A of CYP2E1, the V_max_ and CL_int_ of both the wild-type homozygote and mutant heterozygote correlated with Cyt b5 levels. Significant correlations between Cyt b5 contents and V_max_ and/or CL_int_ of CYP2E1 -333TT (-333T>A) and -352AA (-352A>G) genotypes were also observed. However, for the CYP2E1 mutant homozygote -333AA and mutant heterozygote -352AG, K_m_ was negatively associated and Cl_int_ was positively associated with Cyt b5 content.

## Discussion

This study is the first to comprehensively investigate Cyt b5 mRNA and protein expression levels as well as the effects of Cyt b5 content on the activities of polymorphic CYP1A2, 2B6, and 2E1 in a large collection of normal Chinese liver samples. The results indicate that up to 12- and 19-fold variation exists in Cyt b5 mRNA and protein levels, respectively, in which gender and smoking significantly contributed to individual variation at the protein level. Moreover, Cyt b5 protein levels significantly correlated with the overall activities of CYP1A2, 2B6, and 2E1 in HLM. However, when CYP activities were assessed by SNPs, the effects of Cyt b5 protein levels on CYP1A2, 2B6, and 2E1 kinetic parameters were found to be SNP-dependent.

Although numerous studies have focused on Cyt b5 in the past, the expression levels of Cyt b5 in HLM samples has rarely been reported. An earlier study about relative contents of Cyt b5 protein in 19 HLM found 9.3-fold variation among samples [9], whereas in a recent study by James *et al*., the relative Cyt b5 protein content only exhibited 5-fold variation among 111 liver samples [3]. Takahashi *et al*. reported a mean Cyt b5 content of 177.9 ± 50.9 pmol/mg in Japanese human liver samples, but that study only assessed 11 samples [10]. In the present study, we measured the Cyt b5 contents in 123 Chinese human liver samples and found that the protein expression was not normally distributed. The median content was 270.01 pmol/mg (mean: 273.40 ± 84.36 pmol/mg), which is higher than that presented in the study by Takahashi *et al*. [10]. In addition, another study measured Cyt b5 contents ranging from 7 to 660 pmol/mg (mean: 320 ± 180 pmol/mg) in 46 Caucasian samples [11], which is higher than the present results. It is possible that these differences are due to the various donor groups or ethnicities used in each study.

To the best of our knowledge, the study conducted by Gan *et al*. is the only one to date that has compared the effects of age and gender on Cyt b5 contents [11]. That study found that mean Cyt b5 contents based on spectrophotometric assessment were significantly lower in the elderly donor group than those of the young donor group, whereas the difference in content between the two groups based on immunoblot did not reach statistical significance [11]. We failed to identify age as an influential factor of Cyt b5 content, but we did find that gender and smoking significantly affected content. Smoking can induce the production of several CYPs, such as CYP1A2 [30], and therefore it is worth exploring whether smoking has a similar effect on Cyt b5.

In this study we also found that Cyt b5 mRNA levels were not normally distributed and displayed considerable variation. Moreover, Cyt b5 mRNA levels did not correlate with protein contents. These results indicate that post-transcriptional regulation might be involved in human liver, which has been suggested by a study showing the miR-223 down-regulates Cyt b5 [10].

Although Cyt b5 has been shown to play an important role in CYP-catalyzed reactions, almost all of the information has been obtained from studies using heterologous reconstitution expression systems, either through the co-expression of Cyt b5 with CYP or the addition of purified Cyt b5 to reconstitution systems. Little is known about the impact of Cyt b5 on CYP activity in the physiological environment. Yamazaki *et al*. inferred that Cyt b5 was essential for modulating CYP3A activity according to the inhibition of Cyt b5 antisera to the metabolism of testosterone in HLM [24], whereas Gan *et al*. reported that variation in Cyt b5 expression in HLMs did not contribute significantly to variation in CYP3A-mediated midazolam hydroxylation [11]. Kaspera *et al*. found that Cyt b5 contents were inversely associated with CYP2C8 activity in HLMs, which was not consistent with published data using recombinant CYP2C8 [31]. However, in the present study, the amount of Cyt b5 protein correlated significantly with the enzymatic activity of CYP1A2, 2B6 and 2E1, suggesting that Cyt b5 affects CYP-mediated catalysis in HLM.

The effect of Cyt b5 on CYP1A2 remains controversial. An earlier study showed that Cyt b5 had no effect on theophylline 8-hydroxylation by CYP1A2 in reconstituted systems [27]. In another recent study by Takahashi *et al*. [10], CYP1A2-mediated 7-Ethoxyresorufin O-deethylation activity was not affected by miR-223-dependent down-regulation of Cyt b5 in HepG2 cells infected with an adenovirus expressing human CYP1A2 [10]. However, our data conflicted with these findings and showed that the level of Cyt b5 protein had a significant impact on CYP1A2 phenacetin O-deethylation activity in HLM. These differences could be due to the substrates used or the source of enzyme complex (HLM in our studies versus recombinant expression in previous studies). In addition, previous studies in reconstituted systems have revealed that Cyt b5 stimulates CYP2B6 and CYP2E1 activities [10,32], which coincided with our study in HLM.

Few studies to date have characterized the effects of Cyt b5 on human CYP polymorphic variants in recombinant systems using engineered mutants, but recombinant protein systems have limitations, including differences in Cyt b5 expression between variants and wild-type or even between batches of the same protein. Moreover, only nonsynonymous mutations in coding regions of CYP have been investigated in recombinant systems. However, most of the nonsynonymous variants of CYP (such as CYP1A2 and 2E1) studied are very rare and common polymorphisms that exist in the promoter, introns, 3’-untranslated region (3’-UTR), and 5’-untranslated region (5’-UTR) as well as heterozygous SNPs also have obvious effects on CYP function. Therefore, a study assessing the impact of Cyt b5 on common polymorphic CYP isoforms may be more meaningful. Palma *et al*. examined the effect of Cyt b5 on the activities of eight naturally occurring variants of human CYP1A2 by coexpressing human CYP together with Cyt b5 in a bacterial cell model and found that Cyt b5 induced CYP1A2 variants to behave more like wild-type [14]. In the present study, 5 SNPs (-3860G>A, -3113G>A, -163C>A, 2159G>A, and 5347C>T) in CYP1A2 were used to assess the correlation between Cyt b5 levels and polymorphic activity of the enzyme. The effect of Cyt b5 on CYP1A2 in HLM varied considerably. Cyt b5 content was positively correlated with the V_max_ and CL_int_ of CYP1A2 wild-types -3860GG, 2159GG, and 5347CC, but had no effect on homozygous mutants -3860AA and 5347TT. In contrast, significant associations between Cyt b5 contents and V_max_ and CL_int_ of CYP1A2 homozygous mutants -163AA and -3113GG were observed, while Cyt b5 had no impact on the activity of wild-type CYP1A2 (Table 2). The *CYP1A2* gene is highly polymorphic, but only a few SNPs are associated with altered CYP1A2 activity [33]. Our study found that all 5 SNPs assessed had no effect on CYP1A2-mediated phenacetin O-deethylation activity. However, the significant relationships observed between Cyt b5 levels and polymorphic CYP1A2 activities in HLM were rather interesting. SNPs -3860G>A and -3113G>A are located in the distal enhancer region of the *CYP1A2* gene. Both -163C>A and 2159G>A are intronic mutations, whereas 5347C>T is a synonymous variant in exon 7 of CYP1A2 [34]. Although -3860G>A, -3113G>A, and -163C>A are considered to have a potential impact on CYP1A2 activities depending upon the substrate and ethnicity [16], none of the 5 SNPs assessed induce a structural change in CYP1A2. Therefore, the varying influence of Cyt b5 on the kinetic parameters of wild-type and variant CYP1A2 is unclear and will require further studies for full elucidation.

SNPs 516G>T and 785A>G in CYP2B6 are exonic mutations associated with amino acid substitutions (Q172H, K262R, respectively) [16,35]. In addition, SNP 516G>T can also lead to incorrect splicing of the CYP2B6 pre-mRNA, which results in a deficient enzyme. It has been shown that these two SNPs can affect drug metabolism and therapeutic efficacy. Our study found that the presence of 516GT and 785AG had significant effects on bupropion 1-hydroxylation compared to the wild-type sequence. Reconstitution experiments using phospholipid vesicles suggest that CYP2B6 requires Cyt b5 for full catalytic activity [36]. In the present study, Cyt b5 was positively associated with the V_max_ and Cl_int_, negatively correlated with the K_m_ of CYP2B6 516 G>T and 785 A>G wild-type genes. However, no association was observed for the variant type enzyme.

Although it is well-accepted that Cyt b5 has essential impact on CYP catalytic activity, the mechanism remains unclear. Some proposed mechanisms include the following: 1) transferring the second electron to CYP; 2) functioning as an allosteric effector; and 3) functioning as a competitive inhibitor of CYP oxidoreductase [37]. The negative correlation between Cyt b5 content and K_m_ of the CYP2B6 516GG and 785AA genotypes suggests that Cyt b5 may physically interact with CYP2B6 wild-type to enhance the binding affinity of CYP2B6 wild-type for the substrate through an allosteric mechanism. As a result, the metabolic rate would increase, resulting in the positive correlation observed between Cyt b5 levels and the V_max_ and Cl_int_ of CYP2B6 516 GG and 785 AA wild-type. However, amino acid changes in the 516 G>T and 785 A>G variants may prevent the interaction of Cyt b5 with CYP2B6, which may be one reason why Cyt b5 has no influence on variant CYP2B6 activity. In reconstituted systems, both holo-b5 and apo-b5 have been shown to stimulate CYP2B6 activity, suggesting that protein—protein interactions may be one of the mechanisms causing enhancement of CYP-catalytic activity by Cyt b5 [7,36,38]. Importantly, this hypothesis is consistent with the findings of the present study.

Compared with other CYP members, CYP2E1 is less polymorphic and common polymorphisms in the *CYP2E1* gene have no important functional impact on its activity [13]. None of the 4 SNPs assessed in this study (-1293G>C, -352A>G, -333T>A, and 7632T>A) had an effect on CYP2E1 activity. However, Cyt b5 levels were positively correlated with the V_max_ and CL_int_ of CYP2E1 SNPs -1293G>C and 7632T>A wild-type homozygote and variant heterozygote. This tendency may be because the -1293G>C and 7632T>A mutations can link together to construct a CYP2E1*5B allele (http://www.cypalleles.ki.se/cyp2e1.htm). Unexpectedly, Cyt b5 not only positively influenced the CL_int_ and/or V_max_ of CYP2E1 -333TT, -333AA, -352AA and -352AG, but also negatively influenced the K_m_ of -333AA and -352AG.

Purified CYP2E1 isolated from liver microsomes of animals has been shown to require Cyt b5 for maximal catalytic activity [23,39]. In reconstituted systems, Cyt b5 has also been reported to stimulate drug-oxidation reactions catalyzed by CYP2E1 [40]. Another study in reconstituted monooxygenase systems has shown that only holo-b5 has the ability to enhance CYP2E1-dependent chlorzoxazone 6-hydroxylation [25], which suggests that Cyt b5 increases CYP2E1 activity by affecting the transferring electron. The inverse relationships between Cyt b5 and K_m_ of CYP2E1 -333AA and -352AG in this study also suggest that Cyt b5 might act as an allosteric modulator for CYP2E1 in HLM.

In conclusion, Cyt b5 mRNA and protein levels varied considerably in a cohort of the Chinese population. Gender and smoking also clearly influenced Cyt b5 content. Cyt b5 had significant impact on the overall activity of CYP1A2, 2B6, and 2E1 in HLM and the effects of Cyt b5 protein on polymorphic CYP1A2, 2B6, and 2E1 activities were SNP-dependent. These findings indicate that Cyt b5 plays an important function in CYP-mediated activities in HLM and may be a contributing factor in the individual variation observed in CYP enzyme activity.
